# Use of pedometer-driven walking to promote physical activity and improve health-related quality of life among meat processing workers: a feasibility trial

**DOI:** 10.1186/1477-7525-11-185

**Published:** 2013-11-01

**Authors:** Suliman Mansi, Stephan Milosavljevic, Steve Tumilty, Paul Hendrick, G David Baxter

**Affiliations:** 1 Centre for Health, Activity & Rehabilitation Research, School of Physiotherapy, University of Otago, Dunedin, New Zealand; 2 School of Physical Therapy, University of Saskatchewan, Saskatoon, Canada; 3 Division of Physiotherapy Education, The University of Nottingham, Nottingham NG5, UK

**Keywords:** Physical activity, Pedometers, Walking intervention, Quality of life

## Abstract

**Background:**

Current evidence supports the use of pedometers as effective motivational tools to promote physical activity and improve health-related quality of life in the general population. The aims of this study are to examine whether a pedometer-driven walking programme can improve health-related quality of life, and increase ambulatory activity in a population of meat processing workers when compared to a control group receiving educational material alone.

**Methods/design:**

A feasibility study employing a randomized controlled trial (RCT) design will collect data at three time points. A sample of up to 60 meat workers will be recruited and randomly assigned to either an intervention group N = 30 (12-week pedometer-driven walking program, brief intervention, and educational material), or control group N = 30 (educational material only). The primary outcomes of ambulatory activity, health-related quality of life, and functional capacity, will be evaluated at baseline, immediately following the 12-week intervention and then at three month post-intervention.

**Discussion:**

This paper describes the design of a feasibility randomized controlled trial, which aims to assess the effectiveness of the introduction of a workplace pedometer-driven walking program compared to normal lifestyle advice in meat processing workers.

**Trial Registration Number:**

(ANZCTR): 12613000087752.

## Introduction

The meat processing industry is the second most important sector in the New Zealand (NZ) economy, employing approximately 24,000 workers, and contributing approximately 13% of New Zealand’s exports [1]. Meat processing involves different work stages that include slaughtering, boning, cutting, and packing, which demand different physical workloads and tasks. These often require prolonged periods in task-related non-neutral postures, potentially leading to an increased risk of occupational injuries.

Work-related disorders and occupational injuries reported to afflict workers in the meat processing industry are varied, with musculoskeletal disorders (MSD) being a commonly reported health problem [2-4]. Relevant risk factors include hazardous working conditions [4,5] with repetitive movements, heavy physical workload, and sustained standing [6-8] that have been linked to increased levels of disability, sick leave, and work incapacity [9]. The prevalence of MSD among meat processing workers has been previously published [2]. For example meat processing in the USA is considered one of the most hazardous industries, with an overall incidence injury rate of 6.9 per 100 fulltime workers in 2009 [10], while in Canada, the incidence injury rate was 23.48 per 1,000 [11]. In NZ the prevalence of MSD is at a higher rate than any other sector based on Accident Compensation Corporation (ACC) claims data in 2005–2006, with annual cost of approximately $12 million [12] while in 2003, the MSD incidence rate for meat processing was 59 per 1000 full-time equivalent workers (FTE) compared to 20 per 1000 FTE for forestry and logging and 16 per 1000 FTE for construction [12]. The most common injuries usually involve the upper extremities [13-15], with shoulder, and neck, as well as lower back, having the highest reported incidence [13,16-23].

These conditions are known to impact on health-related quality of life, increase healthcare costs, and also decrease daily activities [24]. It has also been reported that adults with MSD have an overall poorer health-related quality of life than the general population reporting no pain [24,25]; this effect is likely to be increased among aging employees due to the health problems that accrue over a working life-span.

Improving general health status by increasing the level and capacity for physical activity (PA) may help lead to a reduction in occupational injury and protect workers from accidents; reduce working hours lost as a result of absence due to illness or injury; as well as reduce the costs of treatment, and claims for compensation [26-28].

Physical activity (PA) plays an important role in the prevention and management of various chronic diseases including sedentary obese, high blood pressure, and cardiovascular disease [29-31], with a reduction in premature mortality and improvement in quality of life [32]. Studies have demonstrated the benefit of increased PA in reducing pain and improving quality of life in workplace populations with MSD [33,34]; levels of PA also correlate with reductions in the risk for premature all-cause mortality across all groups and sexes in the general population [35,36]. A critical review by Propper and colleagues identified clear evidence for the benefit of worksite PA to manage MSD among employees [37]. Currently, there are studies that support the use of walking-based interventions that encourage people with MSD (including LBP, osteoporosis, hip, and knee osteoarthritis [38-42] to assume a physically active role in their recovery. A recent publication has also demonstrated the effect of pedometer-driven walking on relieving musculoskeletal symptoms (for both function and pain) in chronic LBP among adults aged 18 or over [43].

National and international PA guidelines have recommended that every adult accumulate at least 150 minutes of moderate-to-vigorous intensity physical activity every week, to gain significant health benefits [44]. Despite the well-known benefits of regular PA, the World Health Organization (WHO) reported in 2008, that 31% of adults over 15 years exhibit a sedentary lifestyle, and have a 20-30% increased risk of mortality compared to active people, with this effect being more noticeable among females compared to males [45,46]. Although The New Zealand Physical Activity Guidelines state that adults should participate in at least 30 minutes of moderate activity on most, if not on all days of the week [47] data from Sport and Physical Activity Surveys NZ 2008 [48] show that only 48.2% of NZ adults are physically activity on five or more days per week, while a recent New Zealand Health Survey 2012 [49], found 54% of adults met the current recommendations of daily PA daily. Physical inactivity is a significant public health issue in New Zealand [45] contributing to non-communicable diseases and health problems; costing approximately $1.3 billion for the 2010 year [50].

Walking is considered to be an ideal form of PA to promote and maintain health status in the general population. For most it requires no additional physical skills, and is achievable by all ages with little risk of injury [51,52]. A systematic review [53] examined the effectiveness of interventions aimed to promote walking that including 19 randomised controlled trials and 29 non-randomised controlled studies. The review concluded that the strongest evidence exists for tailored interventions that focused at the level of the individual needs and sedentary groups such as pedometers with individual goal setting to be more effective to promote walking.

Walking with a pedometer as the intervention tool is becoming widely used in different health related domains to promote PA levels, and improve health status in a wide range of populations. Pedometers can supply valuable information on the number of steps and distance travelled, time spent in an activity, and also provide an estimate of energy expenditure [54]. The majority are a reliable and valid device for increasing and measuring physical activity, particularly as part of a walking programme [55]. In clinical studies pedometers have been widely used in the assessment and management of PA within a range of conditions including sedentary obese [56,57], diabetes [58], and knee osteoporosis [39,59], with an aim to encourage increased habitual physical activity, and improve health-related quality of life.

A variety of workplace pedometer walking interventions have been developed to improve health-related outcomes and increase the PA levels of employees [60,61]. Workplace pedometer-based interventions with goal setting (such as 10,000 steps per day) and weekly e-mail messages have shown a positive effects on PA and health outcomes in the short-term [62,63] as well as long-term [64]. Correlations between number of steps and health outcomes have been shown [60,61,64]: for example Chan and colleague [65] reported in group of intervention an average daily step count increase of 3,451 ± 2,661 with a concurrent significant decrease in body mass index and waist girth. These results illustrate the potential that the workplace offers as an ideal setting for health promotion and PA strategies.

To our knowledge, no study has employed pedometer-driven walking as a motivational strategy and intervention together with goal setting in order to increase daily ambulatory activity among meat processing workers. The meat industry has substantial economic importance to the New Zealand economy. However it is known that the population of meat-workers in New Zealand is aging and has health related issues consistent with an aging population, a sedentary lifestyle, and chronic disease that include obesity, hypertension, diabetes, and other cardiopulmonary problems [66,67]. Although these factors can impact adversely on work productivity and sick leave [9], they are also known to positively respond to increased PA [29,30]. A healthier more active work force will likely be associated with reduced sick leave, reduced injury rates, and increased productivity [60]. The use of a simple, cheap, performance driven physical intervention, and one that is socially interactive, may be a significant step towards improving the health and lifestyle of these workers.

The primary aims of this study are to examine whether a pedometer-driven walking programme, incorporating a brief intervention, along with an educational material can improve health-related quality of life, and increase ambulatory activity in a population of meat processing workers when compared to a control group receiving educational material alone. Secondary effects on blood pressure, body mass index, body fat percentage, and waist circumference will be also measured**.** We hypothesize that the pedometer-driven walking intervention will be a feasible and effective tool to increase participants’ daily ambulatory activity levels and improve health outcomes compared to a control group.

## Methods/design

### Study design

This will be a feasibility study using a randomized controlled trial (RCT) design. Data will be collected at three time points (baseline, 12 weeks (at conclusion of intervention), and 3 months post intervention). The study will recruit up to 60 participants and comprise two arms (i) pedometer-driven walking (PW) n =30), (ii) control group receiving normal lifestyle advice (CG n = 30).This randomised clinical trial will be reported according to the recommendations of the CONSORT statement [68].

### Ethical approval

The study design has been approved by the Otago Human Ethics Committee number (12/313) and the study protocol is registered on the Australian New Zealand Clinical Trials Registry (ANZCTR): 12613000087752. Written informed consent will be obtained before participants enter the study.

### Description and selection criteria of participants

A large, local meat processing plant in the South Island of New Zealand (with 900 people working in 12 departments) has agreed to participate in the study. Employee recruitment will be through advertisements (posters) in different work-sites including the health clinic, plant administration, cafeterias, and all department notice-boards until a target sample (n = 60) is achieved. Participants will eligible to participate if they are: currently working, male or female aged 18–65 years, have a sedentary lifestyle and/or low levels of physical activity (less than 7,499 steps per day); are able to walk continuously for at least 10 minutes; able to read and sign an informed consent form and questionnaires, and are willing to participate for the full study duration.

### Screening

Potential participants will initially be screened for eligibility for entry into the randomized control trial by wearing the pedometer (Yamax Digi-walker SW-200) for seven consecutive days. Participants will be instructed on how to wear and use the pedometer at the assigned location on a waist band above the lateral hip during all waking hours, except for periods immersed in water (bathing, swimming), during certain sporting activities (playing basketball or soccer, etc.), or in bed at night. They will be instructed to reset the pedometer to zero at the beginning of each day, and remove it at the end of each day, record on a step calendar the date and the time pedometer was attached and also removed, and the total number of steps displayed on the pedometer at the end of each day.

Participants who have accumulated an average of 7,500 or more steps per day will be excluded before baseline assessment. In addition, the ability of the participant to be physically able to participate in walking program will be screened using the physical activity readiness questionnaire (PAR-Q) [69]: if a participant answers yes to one or more questions on the PAR-Q, they will be advised to consult their healthcare provider and that physician consent will be required prior to program participation.

### Randomization

**Figure 1:**
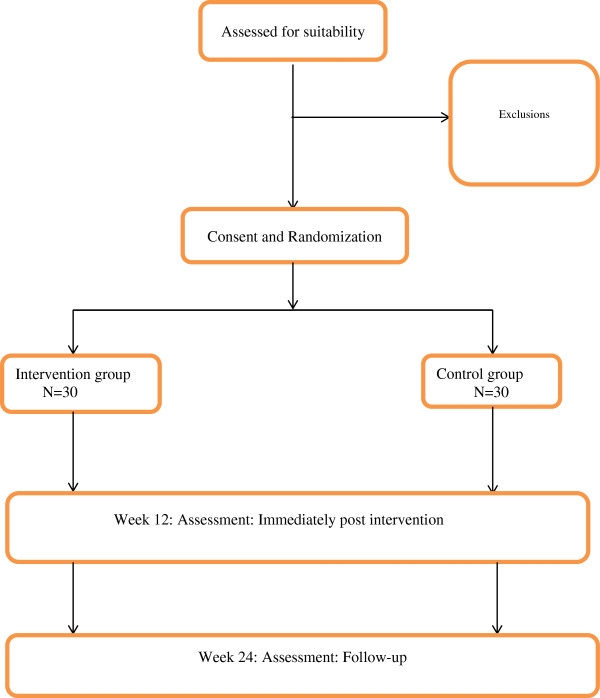
Trial protocol.

After successfully completing the baseline assessment and signing the informed consent form, randomization to one of the two groups will be performed using sealed envelopes. Participants will be invited to choose an envelope from a basket containing envelopes that allocate 50% of the sample for the intervention and the other 50% for the control groups: each will contain the group name for allocation, and the timetable of the study. Researchers and participants will be not be blinded to group allocation. The assessor for final outcome measurements will be blinded to group allocation until the final assessment is achieved. The flow of participants through the recruitment process and randomisation is presented in Figure 1.

### Outcome measurements and methods

The outcome measurements will be made at baseline, immediately after the 12 week pedometer-driven walking programme (and equivalent for control group), and at the 3 month follow-up time point. Data from all outcome measures will be securely stored and only analysed once the trial is complete. The primary outcome measurements are quality of life, PA level, and functional capacity. Secondary outcome measures will include blood pressure (BP), body mass index (BMI), body fat percentage (BF), and waist circumference (WC).

#### ***Health-related quality of life***

This will be measured using The Short Form 36 version2 (SF-36v_2_) questionnaire. The SF-36v_2_ has been widely used to measure quality of life in general and specific populations [70,71]. It has eight domains of health-related quality of life: physical functioning, role limitations resulting from physical health problems, bodily pain, social functioning, general mental health, role limitations resulting from emotional problems, vitality, and general health problems. The SF-36 questionnaire has been validated and is a reliable measure of physical and mental health that can be completed in five to ten minutes [71,72].

#### ***Ambulatory activity levels PA***

Objective change in PA level will be measured using a pedometer. The Yamax Digi-walker SW-200(Yamax, Tokyo, Japan) will be used in this study. This model demonstrates acceptable reliability for research purposes in the adult population [73,74]. It was found to be the most accurate pedometer in counting steps, recording between 1-3% error within both free living and controlled laboratory settings [75-77]; it has been found to most accurately record at walking speeds of 80–107 m^.^min^-1^[78,79]. Each participant will also complete the International Physical Activity Questionnaire short form (IPAQ-SF), which was developed as an instrument to measure health-related physical activity in work age populations, and is a valid and reliable measure for monitoring population levels of physical activity [80-82]. The questionnaire consists of 7 items which provide information within various intensity levels including aerobic activities vigorous-intensity, cycling activities moderate-intensity, walking activities, and sitting time in the last seven days [80,83].

#### ***Functional exercise capacity***

Functional exercise capacity will be measured using the Six Minute Walk Test (6MWT). The 6MWT is a self-paced task that has been used to assess functional exercise capacity within a variety of chronic conditions, as well as in healthy adults [84,85]. It is a practical and simple test which does not require expensive equipment or advanced training for technicians, and only requires a 100-ft walkway. The test involves asking people to walk the longest distance possible on an over-ground, hard surface in a period of 6 minutes. It has been shown to have good reliability and validity when used to assess functional capacity [86,87]. These walks will be performed on a smooth level surface in an under-cover area, using a straight 30 meter line marked off in 5 m segments by a piece of adhesive tape. Technicians will encourage participants with standardized statements such as You’re doing well, Keep up the good work, and do your best, and the total distance walked will be recorded in meters. Percent predicted values for 6MWT will be calculated using the regression equation described by Enright & Sherrill [88].

#### ***Physical activity self-efficacy scale***

The five point scale of Self-efficacy will be administered to assess participants’ beliefs or their confidence in their physical ability to successfully achieve their goals in different situations [89]. The questionnaire consists of 5 questions and response should take no longer than one minute which scored by summing a 5 point Likert scale ranging from (1 = not at all confident to 5 = extremely confident) with a higher score reflecting greater self-efficacy for exercise. This scale has been shown to have acceptable two week test-retest reliability (0.90) and an internal consistency coefficient of 0.78 [89].

#### ***Anthropometric and physiological measures***

During baseline, 12-week, and follow-up 3 months assessments, several secondary measures will be obtained in both intervention and control groups including blood pressure (BP), body mass index (BMI), body fat (BF), waist circumference (WC), height and weight.

Blood pressure will be measured with an Omron MX3 Plus Blood Pressure Monitor (HEM-7200-E) [90] on 3 occasions with a rest period of one minute between measurements.

Body Mass Index (BMI) will be calculated as recorded mass divided by height squared.

Body Fat Percentage will be formulaically measured using skinfold thickness (The Harpenden Skinfold Caliper W/Software) which was taken from four sites (triceps, biceps, subscapular, and suprailiac) according to recommended locations and technique [91,92]. Three measurements to the nearest 0.1 mm will be taken averaged then will be recorded on the report survey. Body fat will be calculated by Linear Software (Durnin and Womersley) [93] which valid for people between 17 and 68 years old: body fat = (triceps + biceps + subscapular + suprailiac skinfolds) according to the participant’s weight kg and age [93,94].

Waist Circumference will be measured using plastic tape by placing it around the waist at the level of the umbilicus (iliac crest).

Height and Weight will be measured without shoes and light clothing using commercially available digital bathroom scales (Terraillon Lovely Classic Electronic Bath Scale) to assess weight which can weigh in 100G increments up to a maximum of 150 Kgs, and a standard laboratory stadiometer (Seca 213 Portable Stadiometer) to measure height.

### Sample size

Sample size calculations for effectiveness will be not performed. As this will be a feasibility study, one of the aims of data collection will be to determine the effect of the intervention which will then allow size calculations for an adequately powered RCT. However, we will invite the pool of ≈ 900 workers from a local meat processing plant on the South Island of New Zealand to consider participating in the study. Volunteer participants will be screened and the study will begin when the first 60 participants who meet eligibility criteria have been recruited (N =30 participants each group).

### Statistical analysis

Statistical analysis will be performed using SPSS software 20.0, and will include descriptive data and means, confidence intervals, and the standard deviation from the mean. This will be determined for all outcome measures recorded at baseline, 12-week, and the follow-up measurement (3 months after the intervention).

The feasibility and acceptably of using pedometers as an intervention to promote ambulatory activity and improve health outcomes in this population will be evaluated through participant satisfaction with the intervention by using survey questionnaire about pedometer usage after completion the intervention. Participants will be asked about participation in the intervention, and about their satisfaction with participation. Details of the questionnaire were described elsewhere [95]. In addition, adherence to the pedometer-driven walking program by using pedometer logs to determine the number of days that pedometer was worn and divided by the total number of intervention days, and a positive change in the outcomes variable over 12-week periods.

## Procedure

### Preparation of participants

All potential participants will be instructed to wear the pedometer on the waistband of their clothing for seven days, based on previous study protocols [96-99] in order to establish baseline step-counts during normal daily activity. Tudor-Locke and Bassett [52] have classified pedometer-determined PA in adults by an average step count of less than 5,000 steps/day as a sedentary lifestyle, and 5,000 to 7,499 steps per day as low activity; therefore, participants who accumulate an average baseline step count of 7,500 steps per day or more will be excluded; remaining participants will be randomized to one of the two groups. Baseline outcomes measures were collected during the seven days pedometer assessment including the primary and secondary outcomes. After randomization, all participants will attend a 30 minute education session on the health benefits of being physically active, then participants in the walking group will receive a brief intervention group session of up to 70 minutes, including a 10 minute self-efficacy walk, a 30 minute session focussing on physical activity behaviour change, and 30 minutes focused on the education resource material (physical activity and PA booklet). This intervention session will be based on the Back2Activity protocols [100] and conducted by physiotherapists (including one with training in motivational interviewing), and student researcher.

### Intervention protocol

The 12 week pedometer-driven walking intervention will be based on self-regulation theory (SRT) [101] and include goal setting, feedback, educational material, and the use of a step calendar for self-monitoring. Participants will be required to walk for at least five days per week to meet evidence based international guidelines that recommend adults to accumulate at least 30 minutes of moderate intensity activity, on at least five days/week, to achieve optimal health benefits [102].

#### ***Educational materials***

Participants in both the walking (intervention group) and control groups will also receive standardised educational material that consists of written and graphical information describing the importance of walking as a PA for health benefits and prevention of disease [29,30].

#### ***Goal setting***

At the beginning of each week, participants will receive a weekly email reminder about his or her step-count goal for that week based on their baseline walking activity level; the goal will aim to gradually increase the level of activities by 5% from their previous goal setting target with an aim to reach at least 10,000 steps per day at the end of the 12 week period. These targets are based on international guidelines for walking interventions [52]. However for those who reached 10,000 steps per day at any time during the program will be also encouraged to maintain and increase their physically active lifestyle.

#### ***Step count and feedback***

Participants in the intervention group will receive permanent step-count feedback by looking at the digital display on the pedometer monitor. Participants will also receive personalized weekly emails about daily average step-count and additional health information, to encourage their adherence with the program.

#### ***Step calendar***

Participants in the walking group will be given a diary to record their walks and note each day as to whether they are adhering to the program, the time of day, duration of the walk, the week’s step-count goal, and the number of steps taken at the end of each day.

### Control group

Participants randomly allocated to the control group will be encouraged to read the educational activity material and be asked to record any exercise they perform over the 12 weeks. At the completion of the 12 weeks, and at the follow up at 24 weeks, these participants will again wear the pedometer for one week to establish a weekly step count for comparison to baseline scores.

## Discussion

Although pedometer-driven walking has been increasingly investigated as a management strategy for chronic disease and workplace populations, there has been no previous study of their use as an intervention for meat processing workers. This study will focus on a walking program as a management strategy for those populations. Results of this feasibility study will be used to inform the development of a future fully-powered controlled trial of the effectiveness of this intervention in this population.

## Abbreviations

RCT: Randomized controlled trial; MSD: Musculoskeletal disorders; PA: Physical activity; PW: Pedometer-driven walking; CG: Control group; SFF: Silver fern farms; BP: Blood pressure; BMI: Body mass index; BF: Body fat percentage; WC: Waist circumference; SF-36v2: The short form 36 version2 questionnaire; IPAQ-SF: International physical activity questionnaire short form; HRQL: Health related quality of life; SW200: The Yamax Digi-walker pedometer SW-200(Yamax, Tokyo, Japan; 6MWT: The Six Minute Walk Test; SRT: Self-regulation theory; NZ: New Zealand; ACC: Accident Compensation Corporation.

## Competing interests

The authors declare that they have no competing interests.

## Authors’ contributions

All authors have been involved in the development of the study design and research protocols. All authors read and corrected draft versions of the manuscript and approved the final manuscript.
